# Comparative Computational and Experimental Detection of Adenosine Using Ultrasensitive Surface-Enhanced Raman Spectroscopy

**DOI:** 10.3390/s18082696

**Published:** 2018-08-16

**Authors:** Emma M. Sundin, John D. Ciubuc, Kevin E. Bennet, Katia Ochoa, Felicia S. Manciu

**Affiliations:** 1 Department of Physics, University of Texas at El Paso, El Paso, TX 79968, USA; emsundin@miners.utep.edu (E.M.S.); jdciubuc@miners.utep.edu (J.D.C.); kochoa2@miners.utep.edu (K.O.); 2 Department of Biomedical Engineering, University of Texas at El Paso, El Paso, TX 79968, USA; 3 Division of Engineering, Department of Neurologic Surgery, Mayo Clinic, Rochester, MN 55905, USA; Bennet.Kevin@mayo.edu; 4 Border Biomedical Research Center, University of Texas at El Paso, El Paso, TX 79968, USA

**Keywords:** surface-enhanced Raman spectroscopy, theoretical calculations, adenosine detection, silver nanocolloids, label-free optical biosensors

## Abstract

To better understand detection and monitoring of the important neurotransmitter adenosine at physiological levels, this study combines quantum chemical density functional modeling and ultrasensitive surface-enhanced Raman spectroscopic (SERS) measurements. Combined simulation results and experimental data for an analyte concentration of about 10^−11^ molar indicate the presence of all known molecular forms resulting from adenosine’s complex redox-reaction. Detailed analysis presented here, besides assessing potential Raman signatures of these adenosinic forms, also sheds light on the analytic redox process and voltammetric detection. Examples of adenosine Raman fingerprints for different molecular orientations with respect to the SERS substrate are the vibrational line around 920 ± 10 cm^−1^ for analyte physisorption through the carbinol moiety and around 1600 ± 20 cm^−1^ for its fully oxidized form. However, both hydroxyl/oxygen sites and NH_2_/nitrogen sites contribute to molecule’s interaction with the SERS environment. Our results also reveal that contributions of partially oxidized adenosine forms and of the standard form are more likely to be detected with the first recorded voltammetric oxidation peak. The fully oxidized adenosine form contributes mostly to the second peak. Thus, this comparative theoretical–experimental investigation of adenosine’s vibrational signatures provides significant insights for advancing its detection, and for future development of opto-voltammetric biosensors.

## 1. Introduction

Adenosine, a nucleoside composed of a purine molecule adenine bonded to a ribose sugar moiety (ribofuranose) [1], serves multiple roles in the regulation of human physiological systems [2,3,4,5,6,7,8,9,10,11,12,13,14,15,16,17,18,19]. These roles include slowing the heart rate, dilating blood vessels and reducing blood-pressure, regulating the sleep-wake cycle, blocking synaptic potentials, and regulating the sympathetic nervous system [2,3,4,5,6,7,8,9,10,11,12,13,14,15,16,17,18,19]. Consequently, adenosine has a long history of use as a therapeutic agent, beginning with its experimental use in the 1930s, when it was first identified and extracted [2]. The ring structure of adenosine was also established at this time using methylation and hydrolysis [2]. Derivatives of adenosine include the phosphorylated biochemical molecules adenosine monophosphate (AMP), adenosine diphosphate (ADP), and adenosine triphosphate (ATP), which are active in energy transfer and cell signaling, including the cyclic adenosine monophosphate (cAMP) pathways. In plants, a related molecule, L-adenosine, acts as a second messenger [3].

Adenosine is prominent in the regulation of the cardiovascular system, and has been extensively studied in this context [4,5,6,7,8]. It also possesses antithrombotic properties. In the early 1960s, a group of extracts named “circulation hormones”, which included adenosine-type substances, were observed to act as vasodilators, to decrease arterial blood pressure and to slow the heart rate [4]. As such, these extracts were applied clinically on an experimental basis to conditions including angina, hypertension, arteriosclerosis, and Raynaud’s disease, although medical opinions varied at the time regarding the therapeutic value of these treatments [4,5,6,7,8]. Later research for cardiac effects of adenosine targeted coronary microcirculation and its importance in pre-conditioned cardiac states and in attenuation of ischemia-reperfusion injuries [5,6]. Potent vasodilator, adenosine affects the coronary micro-vessels, resulting in a hyperemic response, which is therapeutically and diagnostically useful. Adenosine was also used for treating paroxysmal supraventricular tachycardia and in diagnosing tachycardia of unknown cause [7]. Consequently, adenosine has been considered for decades to be the gold standard for the diagnosis of cardiac diseases [4,5,6,7,8].

In addition to its major role in cardiac physiology, diagnosis, and therapy, adenosine also has important roles in the physiology of learning, memory, and sleep [9,10,11]; in the pathophysiology of brain disorders such as Parkinson’s disease, drug addiction, and depression [12,13,14,15,16]; and in the regulation of anti-secretory effect in the stomach, of anti-epileptic effect, and of renal functions [17,18,19]. Furthermore, because adenosine is a product of ATP degradation, its release from cells can also be correlated with a high metabolic rate or metabolic stress [20]. The extracellular concentration of adenosine in the brain has not only been linked to sleep regulation through slow wave activity [9], but has also been shown to modulate it [10,11]. In the nervous system, adenosine regulates sympathetic nervous system activity [5], and modulates the release of neurotransmitters such as acetylcholine, glutamate, *gamma*-aminobutyric acid (GABA), and dopamine [12]. An inhibitory neurotransmitter, adenosine, selectively blocks certain synaptic potentials in the cortex of the cerebellum, and, at concentrations between 5 and 100 μM, decreases the presynaptic release of neurotransmitters at excitatory synapses [14].

Due to the importance of this bioanalyte, many studies have been reported using different approaches to its investigation [3,4,5,6,7,8,9,10,11,12,13,14,15,16,17,18,19,20,21,22,23,24,25,26,27,28,29,30,31,32,33,34]. The most commonly employed method used to characterize the physiologically relevant forms of adenosine is based on electrochemical detection [13,22,23,24,25], which was initiated by Dryhurst [21]. Since the electrooxidation peak of adenosine occurs at a high positive potential of 1.4 ± 0.1 V (i.e., of 1.3 V using slow scan cyclic voltammetry vs. Ag/AgCl [21] and about 1.4 V using carbon-fiber electrodes and fast-scan cyclic voltammetry [13,22,23,24,25]), there is always a high probability that this peak overlaps with the discharging potentials of supporting electrodes. To overcome the problem of distinguishing between the signal current and the background discharge current, different types of electrodes with different surface chemical functionalization have been developed, such as carbon-fiber and boron-doped diamond-based electrodes [13,22,23,24,25]. Microdialysis sampling, another method that has been used for detection of chemical changes of bioanalytes, has a lower temporal resolution, and introduces a greater chance of tissue damage due to the dimensions of the relatively large probes employed [26,27].

As alternatives, recent research has also considered optical approaches for accurate and sensitive adenosine detection, such as those employing UV-VIS absorption or those centered on surface-enhanced Raman spectroscopy (SERS) [28,29,30,31,32,33,34]. Unfortunately, the accuracy of tracking adenosine with UV-VIS absorption, which has a signature at 260 nm, was reported to be quite low due to interference of other bioanalytes in the same optical window [28]. However, in view of the importance of this purine nucleoside in human physiology, the literature in SERS investigations of adenosine is surprisingly limited [29,30,31,32,33,34]. The literature employing comparison of SERS experimental vibrational spectra of adenosine with quantum chemical calculations is even scarcer [34]. Therefore, the work presented in this study aims to shed more light on ultrasensitive detection of adenosine through comparison of SERS experimental results obtained at concentrations and in time frames characteristic of physiological processes [32] with computational investigations based on density functional theory (DFT). In this way, not only is a better understanding of different configurations of adenosine molecule in the vicinity of metallic silver nanoparticles achieved, but the associated vibrational changes discussed here contribute an accurate assessment of label-free detection of adenosine, which is extremely valuable for the future development of biosensors [35].

## 2. Experimental and Computational Methods

The sample preparation of the silver nanoparticles (Ag NPs) used as SERS substrates and their associated characterizations regarding size distribution, which varied from 5 nm to 20 nm, were reported elsewhere [32]. The adenosine (C_10_H_13_N_5_O_4_, >99%) was purchased from Sigma-Aldrich (Milwaukee, WI, USA) and used “as is.” Aliquots of 90 μL of water-dispersed synthesized Ag NPs were mixed with 10 μL of diluted adenosine solution, for a final concentration of about 10^−11^ M of adenosine in the mixture. To increase the sample uniformity, as well as the probability of the analyte, at this very low concentration, to be in the proximity of silver nanoparticles, the 100 μL mixture was sonicated for 20 s before drop-casting on clean cover slips.

An alpha 300 RAS WITec system (WITec GmbH, Ulm, Germany), with excitation by a 532 nm frequency-doubled neodymium-doped yttrium–aluminum–garnet (Nd:YAG) laser was used for data acquisition performed at ambient conditions and in a backscattering geometry. To avoid sample damage, the power output of the laser was restricted to about 100 μW for SERS measurements. Also, a 20 X long-focus objective (Olympus, Tokyo, Japan) was used for focusing. A higher power, of a few mW, was employed in the measurement for standard adenosine powder. The fast acquisition of Raman SERS spectra was performed using time series measurements, at 500 ms per spectrum. The WITec Control 1.60 software with its fast data acquisition capability was employed for such measurements.

A Gaussian-09 analytical software suite was used for the current quantum chemical density functional calculations. The Raman vibrational frequencies of an energetically and geometrically optimized adenosine molecule were obtained using Becke three hybrid exchange [36] and the Lee-Yang-Parr correlation functional, B3LYP [37]. While a Pople split valence diffused and polarized 6-311++G(d,p) basis set were used for analysis of adenosine molecular forms, an LanL2DZ basis set, which takes into consideration the pseudopotentials for metal atoms, was used for SERS calculations applied to adenosine in the proximity of silver dimers. Parsing of the simulated Raman output data with an in-house algorithm developed in C++ utilizing the Qt framework, and subsequent conversion through MATLAB version r2016a, were also performed. Furthermore, to enable easier comparison of Raman calculated vibrations with experimental results, a conversion of Raman activities into relative Raman intensities [38,39], a normalization of Raman peak intensities by a factor of f = 1 × 10^−12^, and an adjustment of Raman peak shapes with a Lorentzian band having a full width at half maximum (FWHM) of 7 cm^−1^, were also done.

## 3. Results and Discussion

**Figure 1:**
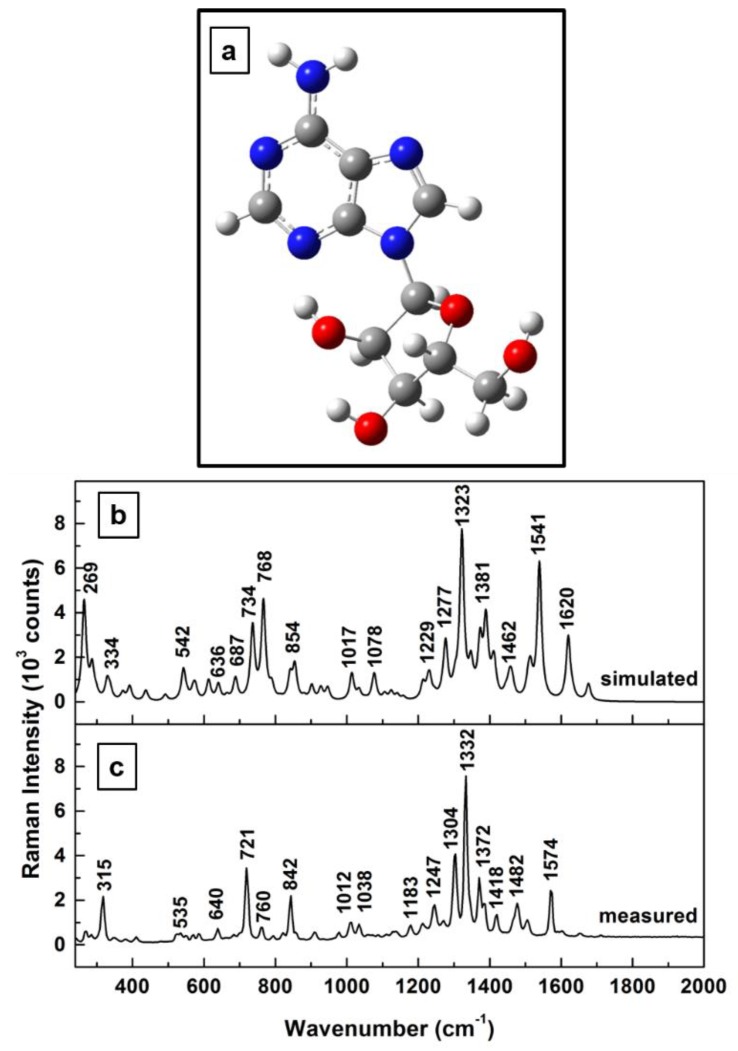
(**a**) Adenosine structural representation in neutral state after energy optimization. Red and blue colors were used for oxygen and nitrogen atoms, respectively. (**b**,**c**) theoretically calculated and experimentally measured Raman vibrations of adenosine, respectively. The Raman spectrum was recorded for the standard adenosine powder.

The adenosine molecule contains 32 atoms and has C_1_ group symmetry. Therefore, 90 possible fundamental vibrational modes are predicted to be active in both infrared (IR) and Raman spectroscopies [34]. We show in Figure 1a–c the energetically optimized structural representation of the adenosine molecule (Figure 1a) together with a comparative analysis of calculated vibrational modes (Figure 1b) and measured Raman frequencies (Figure 1c). Our theoretical calculations, which are comparable to those recently reported by Bakkiyaraj et al. [34], agree relatively well with the experimental results, with discrepancies in vibrational frequency locations, on average, of 12 ± 3 cm^−1^. A scaling factor of 1.01 was used for this set of our simulated frequencies. Sources of these discrepancies could be the force field constants employed in quantum mechanical approaches [38,40], as well as the molecule’s flexibility, with its known configurations in either syn (closed) or anti (open) forms (i.e., changes in the orientation of the furanose ring with respect to that of the adenine ring through the C–N glycosidic bond) [34].

**Figure 2:**
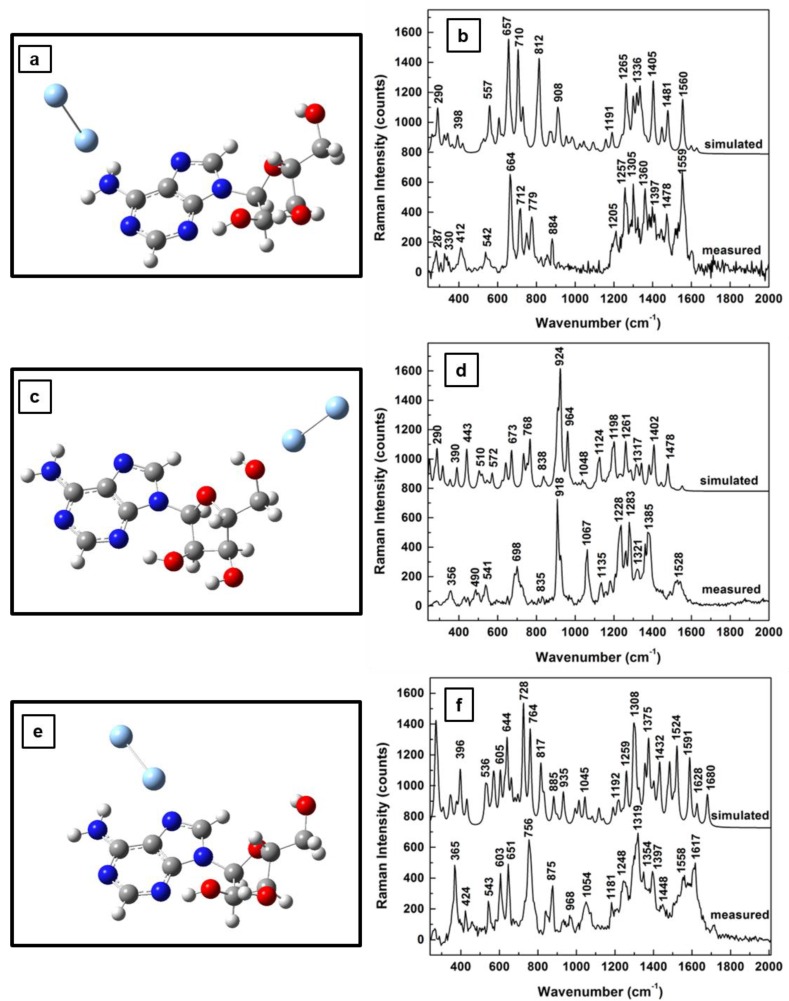
(**a**,**c**,**e**) Adenosine structural representation after energy optimization for silver dimer in the proximity of the NH_2_ site of the adenine ring, in the vicinity of the carbinol moiety, and between the OH and NH_2_ chemical bonds of the adenine and ribose structural units, respectively. (**b**,**d**,**f**) theoretically estimated and experimentally recorded Raman vibrational spectra of neutral adenosine associated with (**a**,**c**,**e**), respectively. The spectra are vertically translated for easier visualization and appropriately labeled.

Since the main focus of this work is to investigate changes in adenosine vibrational modes due to the SERS environment used for analyte detection at very low concentrations, the theoretical and experimental results of this effect on Raman frequencies for adenosine’s standard, neutral form are presented in Figure 2a–f. The new energetically optimized structural representations of the molecule in the close proximity of Ag NPs are shown in Figure 2a,c,e, for different positions of the silver dimer. While an energy minimum is obtained when the silver dimer is perpendicularly orientated with respect to the adenine plane and slightly tilted away from the NH_2_ group of the pyrimidine ring (Figure 2a), a quasi-perpendicular orientation is observed for the case of the silver dimer in the vicinity of the carbinol group (Figure 2c). When the silver dimer is positioned between the NH_2_ group and the hydroxyl groups of the ribose structure, a minimum energy configuration occurs for its quasi-coplanar orientation with the adenine (Figure 2e). For each of these silver dimer positions, the corresponding Raman spectra comparing the theoretically predicted and experimentally obtained results are presented in Figure 2b,d,f, respectively. Scaling factors with values varying from 0.93 to 0.97 were used to adjust the computed data with the measurements obtained. Also, the spectra are vertically translated for easier visualization and appropriately labeled.

In addition to a relatively good agreement between the computed and the experimental results, definite vibrational changes are observed in these Raman spectra for these different dimer positions. For example, there are intense Raman peaks in the frequency regions between 650 and 900 cm^−1^ and from 1200 to 1600 cm^−1^ for the adenosine interacting with silver through the NH_2_ and CH sites (Figure 2b,f). Just a dominant vibration around 920 cm^−1^ is seen when the analyte is physisorpted to the metallic surface through the OH functional groups of the ribofuranose moiety (Figure 2d). For the silver dimer positioned between the NH_2_ and the hydroxyl groups (Figure 2f), an increase in Raman activity through a multitude of peaks is observed in comparison with that in Figure 2b. 

Although in all the above cases hydrogen bonds are involved in the analyte interaction with the SERS metallic environment, a potential reason for all the observed silver dimer orientations could be the induced change in the internal polarizability of the molecule (i.e., the internal dipole moment). Supporting considerations are the modifications in the furanose ring orientations, as well as those of the overall ribose configuration. Such modifications also imply anticipated changes in the positions and intensities of vibrational lines that are indeed observed. For instance, when the overall induced molecular dipole moment results from the normal component of the electric field associated with the light excitation, dominantly in-plane vibrations are expected [41,42], such as those observed in Figure 2b, which are mostly associated with adenine ring bending. On the other hand, if the tangential field component induces the molecular dipole moment, out of plane vibrations such as those seen in Figure 2d are likely. Mixed Raman bands are expected for cases in which a normal field is exciting a dipole with a strong component parallel to the metallic surface (see Figure 2f).

**Figure 3:**
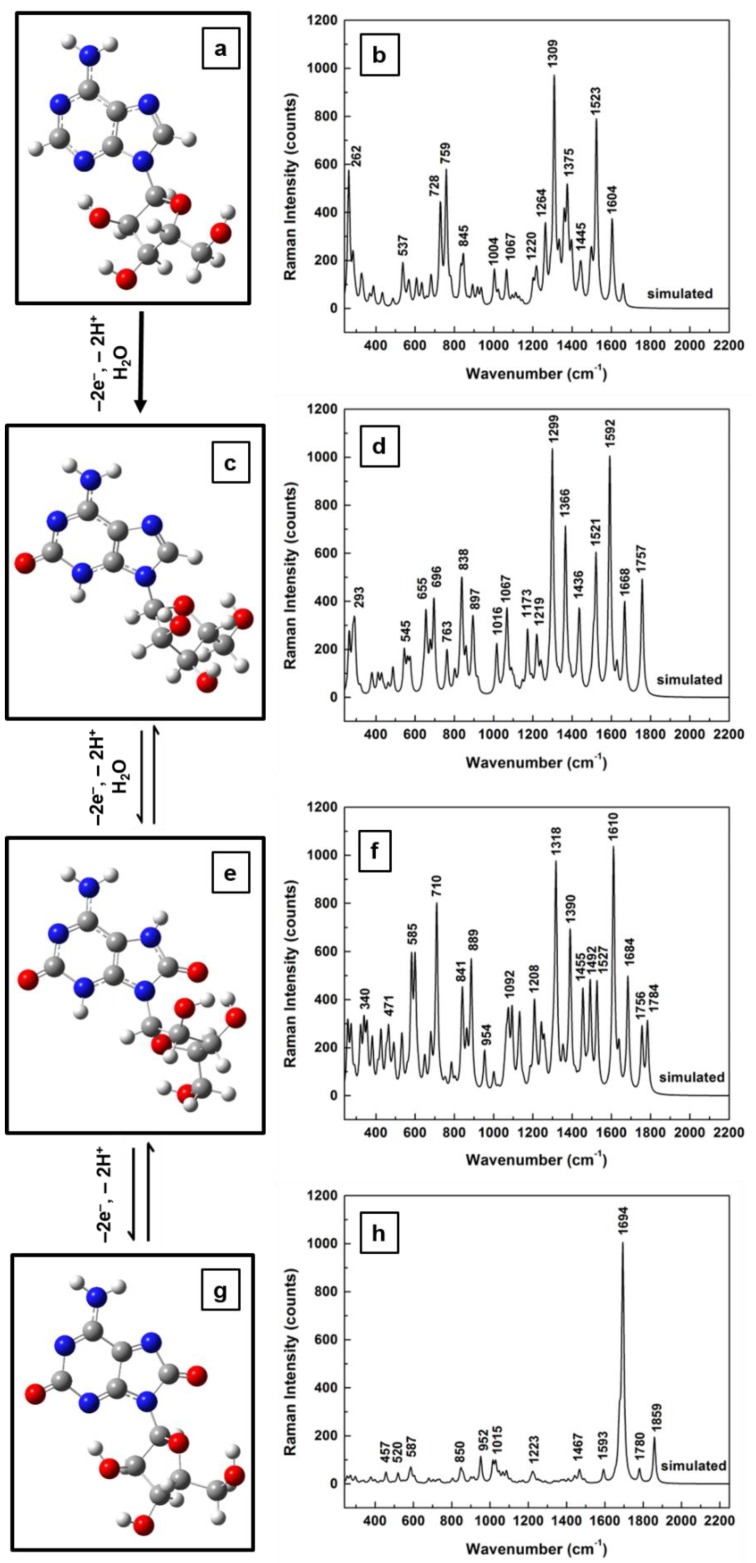
Structural representations of the sequential oxidation steps of the redox process of adenosine consisting of: (**a**) standard, neutral form; (**c**) result of first step of 2-electron oxidation; (**e**) result of second step of 2-electron oxidation, and (**g**) result of last step: fully oxidized adenosine. (**b**,**d**,**f**,**h**) Computed Raman vibrational spectra associated with (**a**,**c**,**e**,**g**), respectively.

Another point worth considering is the greater complexity of the analyte’s redox reaction than those of other neurotransmitters such as dopamine and serotonin [23,25,41,43]. Also, in view of the fact that current sample preparation involves dilution of adenosine in ultrapure water, oxidized forms of adenosine are very likely to occur and to contribute to Raman experimental results. From the perspective of electrochemical detection of adenosine through voltammetry, their contributions were already reported and discussed, such as the presence of two oxidation peaks and that of an undetectable reduction peak [23,25]. Not only does the first oxidation peak occur at a rather high oxidation potential (~1.4 V), but the second peak at 1.0 V cannot be detected unless the first one occurs, suggesting a slow, sequential process of adenosine oxidation [25]. Thus, investigations of Raman vibrational modes of such adenosinic forms that result from known three-step 2-electron molecular oxidation processes are presented in Figure 3a–h. No scaling factors were applied in these cases.

Again, obvious deformations and reorientations of the furanose ring and of the overall ribose molecular configuration are observed in the structural representations of the adenosine oxidative processes, which are shown in Figure 3a (for standard, neutral form), Figure 3c (for the result of the first step of 2-electron oxidation), Figure 3e (for the result of the second step of 2-electron oxidation), and Figure 3g (for fully oxidized adenosine). While a quick look reveals similarities between theoretically obtained Raman spectra for the standard molecule (Figure 3b) and for the first two oxidation forms of adenosine (Figure 3d,f, respectively), there is a visible difference in Figure 3h for the computed Raman spectrum of fully oxidized adenosine (i.e., the result of the last oxidation step). Only a characteristic Raman signature at 1664 cm^−1^ is seen in this last spectrum. A closer, more detailed look at these spectra also reveals an increase in the number of vibrational lines for the first two cases of the 2-electron oxidation steps, an increase that can be associated with the modifications of the ribofuranose configuration (see Figure 3a in comparison with Figure 3c,e). In this context is worth pointing out that, although there is a strong morphological change between the Raman spectra of the standard and of the fully oxidized adenosine, no such conspicuous variation is observed for the ribofuranose structure in these two cases. This suggests a relaxation of the ribose configuration to a form and an orientation that could be described as closer to the original one, despite the molecule’s chemical changes during the intermediate oxidative steps. It may also contribute to explaining the existence of a second oxidation peak in voltammetry and the difficulty of detecting the adenosine reduction peak, as well as the analyte’s slow oxidative process. Other differences between the Raman spectra shown consist of some variations in vibrational line intensities and of their continuous increase in frequency with oxidation, to finally result in a full oxidation fingerprint at 1664 wavenumbers.

**Figure 4:**
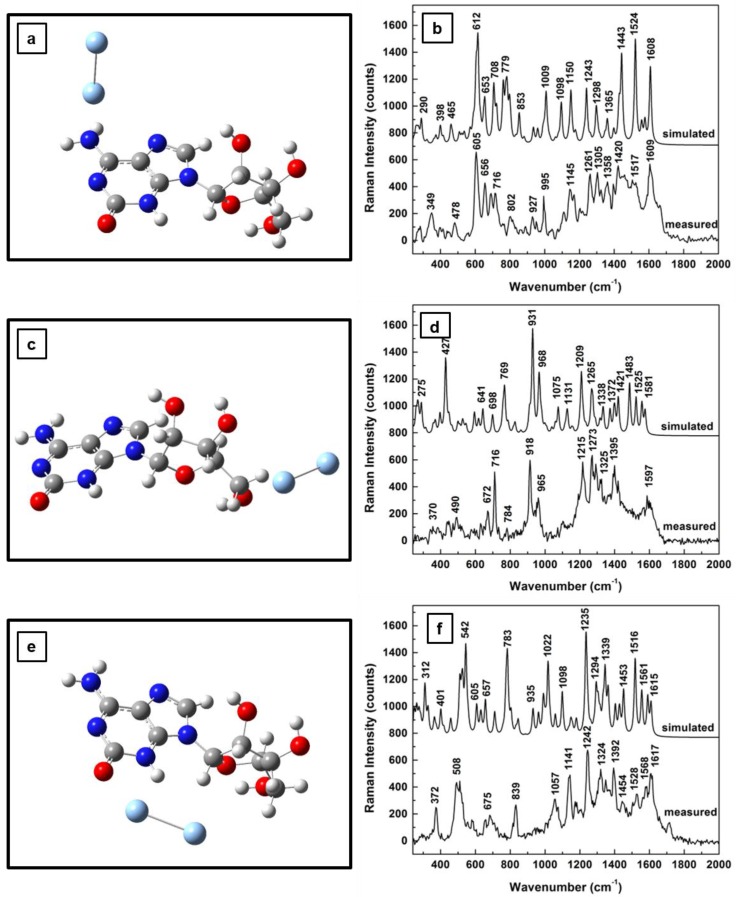
Structural representations of the first step of 2-electron oxidation of adenosine in the proximity of a silver dimer: (**a**) dimer in the proximity of NH_2_ site of adenine ring, (**c**) dimer in the vicinity of carbinol moiety, and (**e**) dimer between the C=O and OH chemical bonds of the adenine and ribose structural units, respectively. (**b**,**d**,**f**) Theoretically estimated and experimentally recorded Raman vibrational spectra associated with (**a**,**c**,**e**), respectively. The spectra are vertically translated for easier visualization and appropriately labeled.

**Figure 5:**
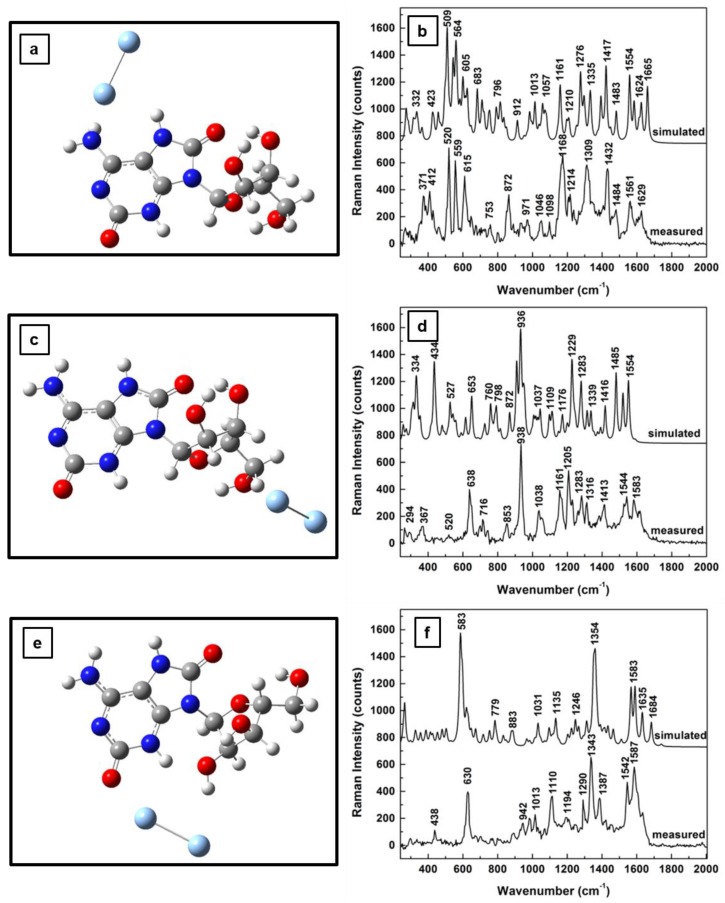
Structural representations of the second step of 2-electron oxidation of adenosine in the proximity of a silver dimer: (**a**) dimer in the proximity of NH_2_ site of adenine ring, (**c**) dimer in the vicinity of carbinol moiety, and (**e**) dimer between the C=O and OH chemical bonds of the adenine and ribose structural units, respectively. (**b**,**d**,**f**) Theoretically estimated and experimentally recorded Raman vibrational spectra associated with (**a**,**c**,**e**), respectively. The spectra are vertically translated for easier visualization and appropriately labeled.

The influence of the silver on the morphological structure of the molecule for the first two 2-electron oxidation steps, together with their computed Raman vibrational lines and comparisons with experimental results, are presented in Figure 4a–f and Figure 5a–f , respectively. While, in Figure 4a, the silver dimer in the vicinity of the NH_2_ group has a perpendicular orientation with respect to the adenine plane, an orientation resembling that of the dimer interaction with neutral adenosine (see Figure 2a), a quasi-planar position of the silver that is almost coplanar with the furanose ring is seen in Figure 5a. There is not much difference in the associated Raman spectra for these energetically optimized molecular configurations for adenosine interactions with the SERS substrate through the NH_2_ site, which are presented in Figure 4b and Figure 5b. Both of these spectra resemble the Raman spectrum in Figure 2b, except for an increase in the number of vibrational lines and a reduction in their intensities at higher frequencies (i.e., 1200–1600 wavenumber region) in Figure 5b. A quasi-planar orientation with the adenine ring is observed in Figure 4c in the case of the first sequential oxidation step of adenosine, when the silver dimer is positioned in the proximity of the carbinol group. A much more tilted dimer orientation is observed in Figure 5c for the second oxidation step of adenosine, at a nearly 45° angle to the adenine plane and coplanar with the furanose ring.

Another interesting observation arises from looking at the predicted Raman vibrations, shown in Figure 4d and Figure 5d, associated with the adenosinic interactions with the silver surface through OH groups. There is a common dominant vibrational line around 920 cm−1 as in the standard adenosine interaction with silver (see Figure 2d). The fact that this vibrational line is commonly seen in all measurements confirms that this configuration is likely to occur. Since this vibration corresponds to in-plane rocking of adenine and furanose rings, as well as to out-of-plane OH and CH bending in the ribose configuration, it also suggests adsorption of adenosine on the silver surface through the lone pairs of oxygen.

Similar coplanar orientations of the silver dimer with the adenine ring are observed for both adenosine oxidation step results presented in Figure 4e and Figure 5e, where the dimer is positioned between the C=O group of adenines and the hydroxyl groups of the ribose. However, much more bending of the ribose configuration towards the silver surface is seen in Figure 5e than that in Figure 4e. Because of this bending the dimer alignment is almost coplanar with the furanose ring in Figure 4e, but oriented perpendicularly to this structural unit in Figure 5e. These last observed differences are reflected in the corresponding Raman spectra that are presented in Figure 4f and Figure 5f through a decrease in the number of vibrational lines in the latter case. Only a few strong vibrations around 600, 1100, 1350, and 1600 cm^−1^ are seen in Figure 5f. This decrease also indicates a more stable morphological configuration of adenosine, which is expected as the molecule continually rearranges to a minimum energy configuration during its oxidative process.

**Figure 6:**
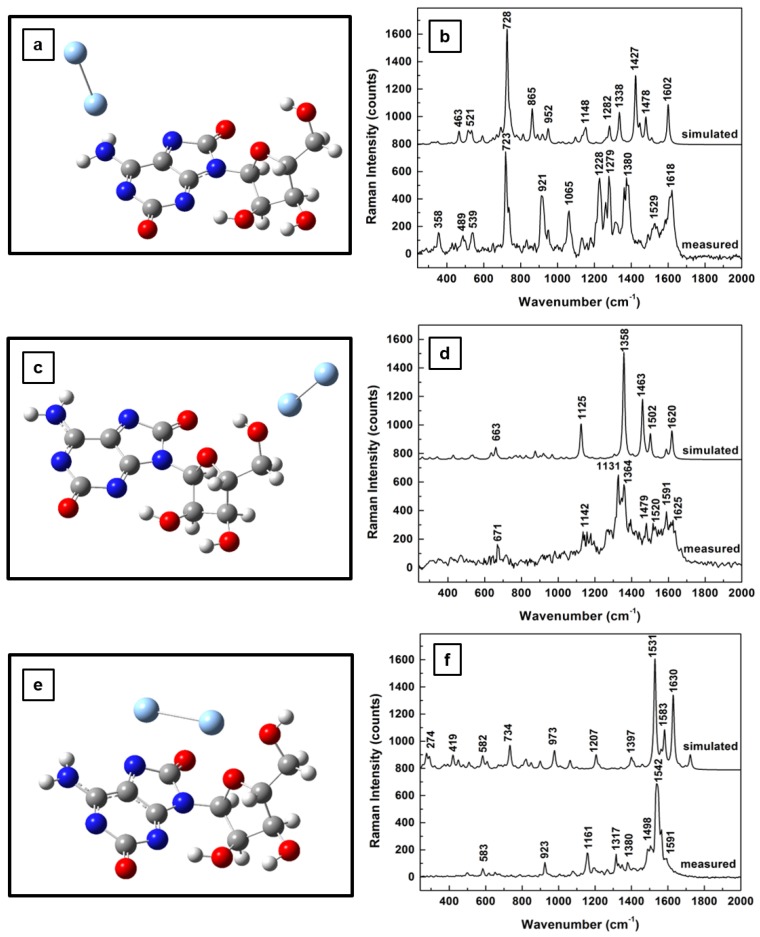
Structural representations of fully oxidized adenosine (the last step of the 2-electron oxidation of adenosine) in the proximity of a silver dimer: (**a**) dimer in the proximity of NH_2_ site of pyrimidine ring, (**c**) dimer in the vicinity of the hydroxyl groups of the ribofuranose moiety, and (**e**) dimer between the OH and NH_2_ chemical bonds of the ribose and adenine structural units, respectively. (**b**,**d**,**f**) Theoretically estimated and experimentally recorded Raman vibrational spectra associated with (**a**,**c**,**e**), respectively. The spectra are vertically translated for easier visualization and appropriately labeled.

A further decrease in the number of Raman peaks for all the silver dimer positions is seen in Figure 6b,d,f, for the fully oxidized molecule in the vicinity of the SERS surface. Dominant signatures around 700, 1350, and 1600 cm^−1^ are observed for the oxidized adenosine interacting with the silver through the NH_2_ group, through the hydroxyl group, and through both of these chemical groups, respectively. A perpendicular orientation of the dimer with respect to the adenine plane is observed in Figure 6a and is similar to those already discussed for the dimer positioned in the vicinity of the NH_2_ chemical group. The evident difference is just in the orientation of the ribose unit. In the case of the molecule’s interaction with silver through the hydroxyl group of the carbinol moiety the dimer orientation, which is presented in Figure 6c, takes after those observed in Figure 2c and Figure 5c for the standard form and the result of the second oxidation step of adenosine, respectively. There is an angle of almost 45° between the dimer and the adenine plane, with the dimer coplanar with the furanose ring. While the co-planarity with the furanose ring is still seen in Figure 6e for silver positioned between the OH and NH_2_ chemical bonds of the ribose and adenine structural units, respectively, as is observed in Figure 5e for the result of the molecule’s second oxidation step, the dimer orientation with the adenine ring still remains quite tilted, at an almost 45° angle.

**Figure 7:**
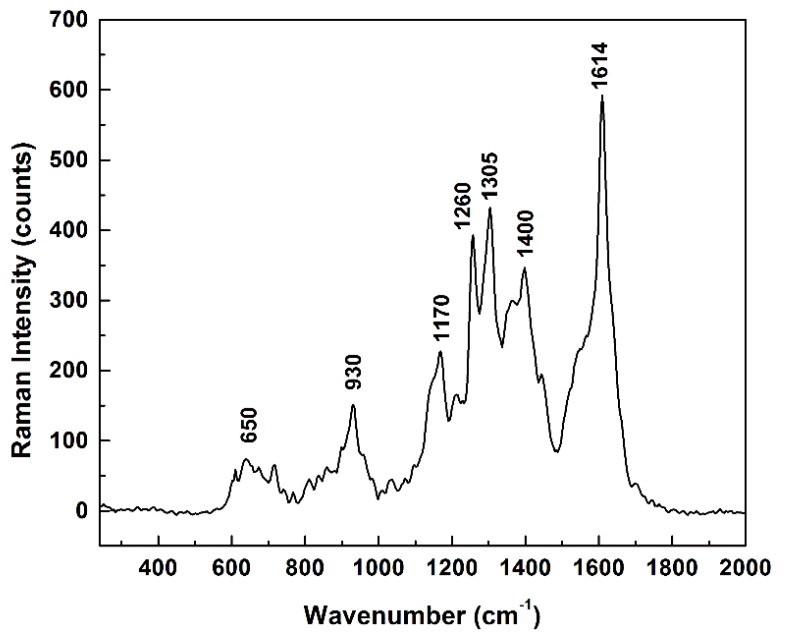
Overall average of 400 Raman spectra recorded in different spots on the sample (8 different time series acquisitions, of 50 spectra each and at 200 ms per spectrum).

All of these changes in dimer orientations with respect to the main structural units of adenosine demonstrate, besides the known flexibility of the molecule, a probable adsorption of the analyte on the silver surface through both hydroxyl/oxygen sites and NH_2_/nitrogen sites. This remark is supported by the long series of SERS measurements of adenosine that are presented in Figure 7 in the form of the average of all the very fast measurements recorded (some not presented here), which shows main Raman features centered around 650, 930, 1170, 1260, 1305, 1400, and 1614 cm^−1^.

The previous observations also reveal a tendency of the molecule to interact with the SERS environment predominantly in its partially oxidized forms combined with its standard form, and somewhat less in its fully oxidized form. This last statement corroborates with the two observed oxidation peaks in voltammetry, as well as with the undetectable reduction peak. If the first voltammetric oxidation peak corresponds to a combination of partially oxidized adenosinic forms and the standard form, and the second voltammetric peak to the fully oxidized adenosinic form, this could explain why there is no reduction peak in voltammetric measurements (besides the reaction irreversibility). It also explains why the second voltammetric oxidation peak cannot be detected unless the first one occurs.

## 4. Conclusions

With the goal of better understanding the detection of adenosine at physiological levels, in this SERS study we presented a comparative theoretical and experimental approach for the analyte at a concentration of 10^−11^ molar. In addition to the standard, neutral form of the molecule, other adenosinic forms that are likely to occur during the analyte complex oxidative process, as well as during sample preparation for current Raman measurements, are also analyzed and discussed here. The positioning of the silver dimer in proximity to different structural parts of the molecule, such as the NH_2_ site of the adenine, the carbinol moiety, and between the hydroxyl/oxygen and NH_2_ sites of the ribose and adenine structures, respectively, reveals a probable adsorption of adenosine on the silver surface through both sites: the known proton donor hydroxyl group and the NH_2_. Although characteristic Raman signatures corresponding to different molecular orientations and interactions with the silver surface are observed in this work, such as the vibrational line around 920 ± 10 cm^−1^ for analyte physisorption through the carbinol moiety and the one around 1600 ± 20 cm^−1^ for the fully oxidized form, it is difficult to determine reliable numerical estimates of the contributions of different analyte configurations to the overall detection. This difficulty is due to similarities in some other adenosine signatures, such as those around 650 ± 50 cm^−1^, 1300 ± 50 cm^−1^, and 1500 ± 30 cm^−1^, as well as to the discrepancies (on average, 50 ± 10 cm^−1^) between theoretically predicted and experimentally measured vibrational frequencies in the Raman results presented here. Despite these anticipated discrepancies, as the simulated data could not perfectly match the experimentally determined ones, there is relatively good agreement between these results, for all the analyzed cases. This statement is especially valid for the most intense vibrations.

The current results also reveal an obvious change in ribose orientations with respect to that of the adenine ring, suggesting states of the molecule in either of the known syn (closed) or anti (open) forms. There is an obvious bending of the ribose structure during the analyte’s interaction with the silver surface through both the OH and NH_2_ sites simultaneously (see Figure 2e, Figure 4e, Figure 5e, and Figure 6e). As a final observation, considering the redox reaction, which is known to take place in electrochemical measurements, contributions of partially oxidized adenosine forms and that of the standard form are more likely to be detected during the first recorded voltammetric oxidation peak. On the other hand, the fully oxidized adenosine form mostly contributes to the second voltammetric oxidation peak. These remarks also corroborate in explaining why the second voltammetric oxidation peak cannot be detected unless the first peak occurs, as well as in explaining the absence of a reduction peak (besides the chemical reaction irreversibility). In conclusion, the comparison of theoretically predicted Raman vibrations with ultrasensitive experimental data that is presented in this work definitely provides valuable information for advancing the detection and monitoring of adenosine, which is needed if future opto-voltammetric devices are envisioned.
